# How Efficacious Are Patient Education Interventions to Improve Bowel Preparation for Colonoscopy? A Systematic Review

**DOI:** 10.1371/journal.pone.0164442

**Published:** 2016-10-14

**Authors:** Jacob E. Kurlander, Arjun R. Sondhi, Akbar K. Waljee, Stacy B. Menees, Cathleen M. Connell, Philip S. Schoenfeld, Sameer D. Saini

**Affiliations:** 1 Department of Internal Medicine, University of Michigan Medical School, 1500 E. Medical Center Dr, Ann Arbor, MI, United States of America; 2 Veterans Affairs Ann Arbor Health Care System, 2215 Fuller Rd, Ann Arbor, MI, 48105, United States of America; 3 Veterans Affairs Center for Clinical Management Research, 2215 Fuller Rd, Ann Arbor, 48105, MI, United States of America; 4 Department of Health Behavior and Health Education, University of Michigan School of Public Health, 3790 SPH I, 1415 Washington Heights, Ann Arbor, MI, 48109, United States of America; University Hospital Llandough, UNITED KINGDOM

## Abstract

**Background:**

Bowel preparation is inadequate in a large proportion of colonoscopies, leading to multiple clinical and economic harms. While most patients receive some form of education before colonoscopy, there is no consensus on the best approach.

**Aims:**

This systematic review aimed to evaluate the efficacy of patient education interventions to improve bowel preparation.

**Methods:**

We searched the Cochrane Database, CINAHL, EMBASE, Ovid, and Web of Science. Inclusion criteria were: (1) a patient education intervention; (2) a primary aim of improving bowel preparation; (3) a validated bowel preparation scale; (4) a prospective design; (5) a concurrent control group; and, (6) adult participants. Study validity was assessed using a modified Downs and Black scale.

**Results:**

1,080 abstracts were screened. Seven full text studies met inclusion criteria, including 2,660 patients. These studies evaluated multiple delivery platforms, including paper-based interventions (three studies), videos (two studies), re-education telephone calls the day before colonoscopy (one study), and in-person education by physicians (one study). Bowel preparation significantly improved with the intervention in all but one study. All but one study were done in a single center. Validity scores ranged from 13 to 24 (maximum 27). Four of five abstracts and research letters that met inclusion criteria also showed improvements in bowel preparation. Statistical and clinical heterogeneity precluded meta-analysis.

**Conclusion:**

Compared to usual care, patient education interventions appear efficacious in improving the quality of bowel preparation. However, because of the small scale of the studies and individualized nature of the interventions, results of these studies may not be generalizable to other settings. Healthcare practices should consider systematically evaluating their current bowel preparation education methods before undertaking new interventions.

## Introduction

Each year, patients in the United States undergo nearly 14 million colonoscopies [1]. These resource-limited, costly, and potentially harmful procedures are mostly performed in asymptomatic patients for prevention and early detection of colorectal cancer (CRC) [2]. The effectiveness of preventive colonoscopy depends on adequate preparation (i.e., cleansing) of the large bowel to allow for visualization of the mucosal surface and detection of adenomas, the pre-malignant lesions that lead to colon cancer [3]. Yet, a large fraction of patients have unsatisfactory or inadequate bowel preparation. A meta-analysis of trials using split-dose polyethylene glycol-based bowel preparations, the current standard of care, found 23% of patients had an unsatisfactory preparation [4] while observational studies yield varying estimates (17–33%) [5–7]. Inadequate bowel preparation leads not only to missed adenomas but also to incomplete procedures, longer procedure times, shorter intervals between procedures, increased cost, and patient inconvenience and harm [7–10].

With the complexity of bowel preparation, patient comprehension of preparation instructions is critical. Lack of comprehension may contribute to non-compliance, a strong patient-level predictor of sub-optimal bowel preparation with split-dose preparations [11]. In one single-center observational study, nearly half of patients undergoing screening colonoscopy reported that they received no verbal instruction and nearly one-third reported that they received no written instruction for bowel preparation [11]. Others have reported that up to one in five patients may not follow recommendations for a split-dose bowel preparation [12].

Multiple studies have evaluated the efficacy of patient education interventions to improve the quality of bowel preparation. These studies have used various delivery platforms; yet, there is no consensus on how best to educate patients before colonoscopy. A recent recommendation from the US Multi-Society Taskforce on Colorectal Cancer recommended that all patients receive both oral and written instructions before colonoscopy but did not specify how or when these instructions should be delivered [13]. With this lack of standardization, endoscopy centers often develop educational materials and processes locally, resulting in significant variation in the instructional content of written bowel preparation instructions [14]. In this context, the aim of this systematic review is to evaluate the efficacy of patient education interventions on the quality of bowel preparation among adults undergoing colonoscopy in prospective, controlled studies.

## Methods

### Study selection

Before commencing the search, an outline of the search strategy was submitted to and approved by PROSPERO, an international prospective database of systematic reviews [15]. We searched CINAHL, EMBASE, Ovid, Web of Science, and the Cochrane Database of Systematic Reviews from database inception to August 2014. CINAHL was used to search the nursing and allied health literature. EMBASE was used to search European literature. Abstracts from meetings of the American Gastroenterology Association, American Society of Gastrointestinal Endoscopy, and American College of Gastroenterology were searched using EMBASE. The search strategy was not restricted by language of the article.

The search strategy for each database was designed in consultation with a health librarian to maximize search sensitivity and was iteratively refined to ensure that all known articles relevant to the topic were captured. The searches combined the terms colonoscopy, patient education/counseling, and bowel preparation, or synonyms of those terms. For example, the Ovid search included the terms (endoscopy OR colonoscopy) AND (purgative$ OR clean$ OR cathartic$ OR cathartics) AND (educat$ OR instruct$ OR teach$ OR counsel$ OR patient education). Details on the search strategies are provided in S1 Table.

We also screened all articles that cited, or were cited by, studies ultimately included in the review. Additionally, we searched clinicaltrials.gov for active clinical trials, and we contacted three content experts who have previously published on this topic for information about potential published or unpublished studies relevant to the review that were not captured by our search strategy.

Studies were evaluated for six inclusion criteria: (1) testing of a patient education intervention, defined as a structured or formal education or counseling intervention intended to improve adherence [16,17]; (2) a primary aim of improving bowel preparation quality; (3) assessment of bowel preparation using one of three validated scales [18–20]; (4) prospective design; (5) inclusion of a concurrent control group, including usual care; and, (6) study participants limited to adults at least 18 years of age. Studies that examined text messaging reminders without additional educational content were not considered educational interventions and were therefore not included. Studies of patient navigation interventions, which are typically multicomponent interventions intended to address individual patient barriers, were also excluded even if one aim was improvement of bowel preparation (since the impact of education could not be separated from the remaining components of the intervention). When the search strategy identified more than one abstract pertaining to the same intervention, a published article (if one existed) or the most recent abstract was used.

All abstracts were screened independently by two members of the study team (JK and AS) to determine whether they met inclusion criteria. If the abstract appeared potentially relevant, the full text article, if one existed, was reviewed to determine whether it met inclusion criteria. For studies published in abstract but not full-text form that appeared relevant, the authors were contacted for additional information. If the author of an abstract was not responsive to questions or did not provide enough information to establish that the abstract met inclusion criteria, it was excluded. Disagreements about inclusion criteria were adjudicated through a consensus process involving a third study team member (SS).

### Data Extraction and Strength of Body of Evidence

For studies published in full text that met inclusion criteria, a structured form was used to extract data on patient sample (size and inclusion/exclusion criteria), study design, study setting (year, location), content and format of both intervention and control, bowel preparation used, and outcomes. When necessary, we contacted authors by email for additional study details. Since the majority of studies reported preparation scores as dichotomous or categorical variables, we contacted the authors of studies that reported only means or medians for additional data. Studies were rated for methodological quality using the Downs and Black scale, which is endorsed by the Cochrane handbook for the assessment of the validity of randomized and non-randomized studies [21,22]. The original instrument included 27 questions. For this review, the instrument was modified in three ways. First, a single question about methodological adjustments for length of follow up was excluded since outcomes in all cases were determined at the time of colonoscopy. Second, a question on whether principal confounders across groups were described not at all (0 points), partially (1 point), or completely (2 points) was defined more specifically (0 points if no confounders were described; 1 point if only age and gender were described; and 2 points if additional variables were described). Third, a question about reporting of adverse events was counted as positive (1 point) if non-attendance at the colonoscopy appointment and/or cancellations were reported since the educational interventions would not be expected to cause clinical adverse events but could conceivably affect appointment attendance. After these modifications, a maximum of 27 points were possible. For abstracts and research letters that met inclusion criteria, only basic study characteristics were extracted given the limited available information.

## Results

### Literature selection overview

**Fig 1:**
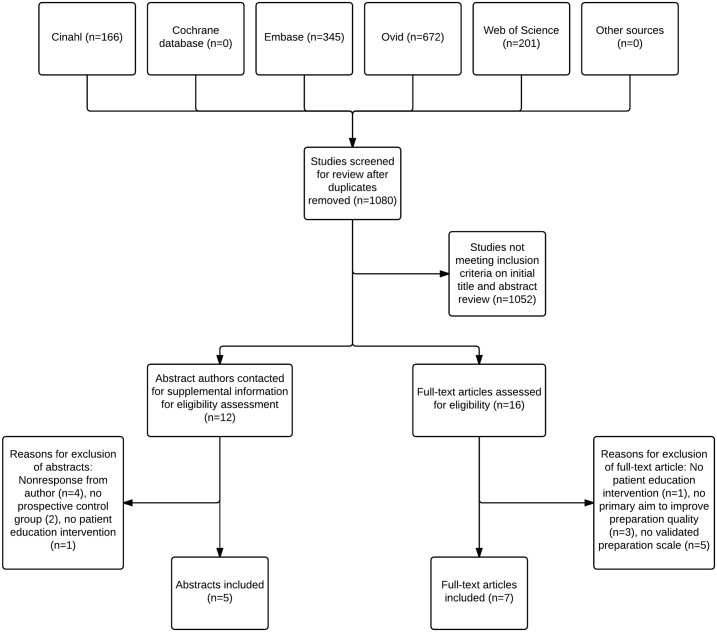
Flow chart for studies identified by literature search.

1,080 unique abstracts were retrieved and reviewed by hand (Fig 1). 16 articles were reviewed in full-text form, five of which were excluded because they did not use a validated preparation scale [23–27], three because they lacked a primary aim to improve preparation quality [28–30], and one because it did not include a patient education intervention [31]. We attempted to contact the authors of 12 abstracts or letters, eight of whom supplied additional information. No additional studies were identified through clinicaltrials.gov or by content experts. Ultimately, 12 studies met our inclusion criteria, including seven full-text articles, four abstracts, and one research letter. No additional studies were identified after hand searching the references of the seven full-text articles, or after searching for articles that subsequently cited any of the seven articles.

### Study characteristics

Characteristics of the seven full-text articles are shown in Table 1. The studies were published between 2011 and 2014 and cumulatively included 2,660 patients. Three of the studies were carried out in the United States [32–34], and four were carried out in Asia [35–38]. All but one of the studies were carried out at a single site [33]. Three of the studies tested educational interventions based on print material (a cartoon visual aid [38], and two illustrated educational brochures [34,39]). Two studies tested educational videos, one delivered online [33] and one delivered in a live group setting [35]. A single study tested telephone calls made by physicians the day before colonoscopy to answer questions and review the content of a previously completed educational session on bowel preparation [36]. Another included a face-to-face educational intervention delivered by a physician in a group setting [37]. The “usual care” control interventions in these studies varied considerably, consisting of some combination of written instructions [33,34,36–39], instructional videos [34], in-person teaching [36,38], question and answer sessions with a health educator [34], and a nurse-staffed call-in line [34]. In six of the seven studies, the treatment group also received the intervention given to the control group [33–37,39]. Only two studies specified the time interval between the educational intervention and the colonoscopy (a factor that could impact efficacy) [34,36]. Three of the seven studies used a split dose bowel preparation [33,37,38]. In all three of these studies, the educational interventions were found to significantly improve bowel preparation quality compared to the usual care condition.

**Table 1:** Descriptions of full-text studies included in review.

	Calderwood, et al, 2011 [33]	Hsueh, et al, 2014 [36]	Liu, et al, 2013 [37]	Prakash, et al, 2013 [34]	Shieh, et al, 2013 [38]	Spiegel, et al, 2011 [35]	Tae, et al, 2012 [39]
**Date, location, setting**	2006–2008, US, single site	2011, Taiwan, single site	2012, China, single site	2011–2012, US, two endoscopy centers	Date not stated, Taiwan, single center	2009, US, single center	2010, Korea, single center
**Treatment intervention**	Visual aid with pictures of adequate and poor preparation, and written message endorsing preparation adherence. Mailed to patients	8-minute instructional video on preparation, diet, and hydration, with pictures. Handout with pictures from film. Question and answer session with physicians	Telephone call with re-education about bowel preparation, timing, and diet done the day prior to procedure. Patients were given a call back number for questions	Online 5-minute video with simplified preparation instructions, pictures of varying preparation quality, and subtitles	Intensive education group with 10 minutes of physician-led education on diet and preparation	Educational booklet designed by health literacy expert based on patient and physician interviews, including illustrations, mailed 1 week before procedure	4-page cartoon visual aid designed as conversation between a patient and provider. Includes pictures of good and poor preparation
**Control intervention**	Standard written bowel preparation instructions in multiple languages	"Routine hospital care"	Instruction from a nurse and written instructions given at a clinic appointment	Instructional brochure	Written instruction, including illustrations and pictures of preparation quality provided by RN at clinic visit	Standard written pharmacy instructions on diet and purgative use, a pre-procedural class, including a 10-minute instructional video, question and answer with a health educator, and phone line to speak with a trained nurse	Verbal explanation by health center staff, written instructions
**Control intervention given to treatment group?**	Yes	Yes	Yes	Yes	Yes	Yes	No
Bowel prep used*	4L PEG +/- bisacodyl	Na phosphate	2L PEG-EL or 90 mL Na phosphate in 1.5 L water, not split dose	Suprep, split dose	Na phosphate, split dose	Na phosphate, Mg citrate, or Moviprep, not split dose	PEG, split dose
**Individual delivering treatment intervention**	N/A	Physician-staffed question and answer session	Physician-staffed phone calls	N/A	Physician	N/A	N/A
**Colonoscopy indication**	Screening	Not specified	Not specified	Not specified	Screening or surveillance	Screening, surveillance, or diagnostic	Screening
**Patients analyzed (n intervention / n control)**	477 / 492	104 / 114	305 / 300	67 / 66	39 / 60	216 / 220	102 / 98
**Inpatient/ Outpatient**	Outpatient	Outpatient	Outpatient	Outpatient	Outpatient	Outpatient	Outpatient

*If not stated in table, the study did not specify whether split dose was used.

PEG (Polyethylene glycol).

The program development phase for the educational interventions was described in three of the seven studies [34,35,39]. Two of the studies explicitly referenced a theory of behavior change (the Health Belief Model) used to develop the interventions [34,39], and one described the involvement of a health literacy expert and psychometrician [34].

### Study quality and risk of study bias

Scores on the Downs and Black scale ranged from 13 to 24 (Table 2 and S2 Table). Five of the seven studies used randomization for allocation [33,34,36,38,39], while the other two used quasi-experimental designs [35,37]. In all cases, the individuals rating the quality of the bowel preparation were blinded to allocation group. Two of the studies used intention-to-treat analysis [34,36]. Either the control or treatment intervention was not completely described in three of the seven articles [33,35,38]. Power calculations were performed in only four of the studies [34,36,38,39].

**Table 2:** Assessment of risk of bias for full-text studies included in review.

Study, year	Allocation method	Endoscopist blinding	Intention-to-treat analysis	Validity score*
Calderwood, et al, 2011 [32]	Randomization	Yes	No	22
Hsueh, et al, 2014 [35]	Quasi-experimental with allocation by week of examination	Yes	No	17
Liu, et al, 2013 [36]	Randomization	Yes	Yes	24
Prakash, et al, 2013 [33]	Randomization	Yes	No	18
Shieh, et al, 2013 [37]	Quasi-experimental with allocation according to physician seen	Yes	No	13
Spiegel, et al, 2011 [34]	Randomization	Yes	Yes	24
Tae, et al, 2012 [38]	Randomization	Yes	No	17

*Based on modified Downs and Black scale, with a range from 0 to 27 [22]. A higher score indicates lower risk of bias.

### Effect on bowel preparation

Six of the seven studies showed a positive effect of the intervention on the quality of bowel preparation (Table 3). The magnitude of effects could not be compared or summarized across studies since there was significant statistical and clinical heterogeneity (e.g, diverse patient samples and intervention formats). This precluded meta-analysis. Studies also used three different bowel preparation scales and reported preparation scores with means, medians, and/or dichotomous categories with proportions. In the one study using the Aronchick scale, the rate of “good” or “excellent” preparations increased by 32% in the treatment group compared to usual care [35]. Among the three studies using the Ottawa Bowel Preparation Scale (in which a lower score is indicative of superior preparation), one study showed that the rate of scores <6 increased by 11% [36]; the two other studies reported their outcomes as medians or means, both with beneficial effects that were deemed statistically significant [33,34]. Among the three studies using the Boston Bowel Preparation Scale (in which a higher score is superior), the rate of scores >5 increased by between 2 and 17%, with a statistically significant difference reported in two of the three studies [32,37,38]. The sole trial that showed no statistically significant effect tested a visual aid depicting bowel preparations of varying quality, and had a high rate of treatment contamination across intervention groups [32]. Supplementary information on preparation outcomes in categorical form could not readily be obtained for two studies [33,34].

**Table 3:** Summary of primary outcomes for full-text studies by preparation scale.

Study, year	Ottawa Bowel Preparation Scale *		Boston Bowel Preparation Scale ^%^		Aronchick Bowel Preparation Scale ^$^	
	Outcome	p-value	Outcome	p-value	Outcome	p value
Calderwood, et al, 2011 [32]			Score ≥ 5: Intervention 91% vs. Control 89%^#^	0.43		
Hsueh, et al, 2014 [35]					"Excellent" or "good" rating: Intervention 81% vs. Control 48%	<0.001
Liu, et al, 2013 [36]	Score < 6: Intervention 82% vs. Control 70%	0.001				
Prakash, et al, 2013 [33]	Median (IQR): Intervention 4 (1–4) vs. Control 5 (3–7)	< 0.001				
Shieh, et al, 2013 [37]			Score ≥ 5: Intervention 97% vs. Control 80%	0.01		
Spiegel, et al, 2011 [34]	Mean (sd): Intervention 4.4 (2.3) vs. Control 5.1 (2.9)	0.03				
Tae, et al, 2012 [38]			Score ≥5: Intervention 93% vs. Control 82%^#^	0.02		

^#^Authors also summarized outcomes with group medians and/or means

*Scale ranges from 14 (very poor) to 0 (excellent)

^%^Scale ranges from 0 (very poor) to 9 (excellent)

^$^Likert scale ranges from “excellent” to “inadequate”

### Non-full text studies

Among the four abstracts and one research letter that met study inclusion criteria (Appendices 3–5), three reported on educational videos [40–42], and two reported on printed instructions presented as a brochure and “photo aids” [43,44]. Four of the five non-full text studies reported a beneficial effect of the tested intervention [40,42–44]. Ergen, et al was the only study in our review performed in an inpatient setting [44]. The one study that did not report a positive effect tested an online educational video, which was used by only 6% of the intervention group [41]. The validity of these non-full text studies could not be assessed.

## Discussion

Colonoscopy is an important preventive, diagnostic, and therapeutic modality, but its effectiveness hinges on successful pre-procedure bowel preparation [13]. When bowel preparation fails, the harms are numerous, including shorter interval repeat procedures, increased expense, missed neoplastic lesions, patient inconvenience, and increased risk of procedural adverse events [7–10,45]. However, the most efficacious manner in which to educate patients about bowel preparation is not known. Our review indicates that multiple different patient education interventions have been demonstrated to improve bowel preparation quality. All but one of the seven published studies resulted in a significant improvement in bowel preparation scores compared to usual care. The single study that failed to show a positive effect tested one of the least intensive educational interventions (a visual aid depicting adequate and inadequate bowel preparations), and had a relatively high rate of treatment contamination [32]. However, these data do not demonstrate the superiority of any one of these strategies over the others. Rather, we can conclude that multiple strategies have the potential to improve bowel preparation. Local factors, such as current bowel preparation education practices, patient characteristics, and staff availability, are likely to affect which intervention is most effective in a given healthcare setting. Before deciding on a specific intervention, healthcare practices might consider rigorously analyzing their current bowel preparation approach, and evaluating the impact of any interventions as part of a Plan-Do-Study-Act (PDSA) cycle [46].

While our review suggests that many educational interventions are efficacious in improving bowel preparation prior to colonoscopy, it also highlights important methodological concerns. Several studies had limited external validity (i.e., generalizability). For example, the study by Liu, et al, of telephone re-education before colonoscopy was conducted in China, where roughly 2/5 of the sample had only completed elementary school [36]. Because illiteracy increases the risk of inadequate bowel preparation, this intervention may be less effective in more educated populations [47]. Prakash, et al, examined the use of an online instructional video but reported 100% adherence in the treatment group (it is unclear if this is per-protocol or intention-to-treat analysis) [33], which is unrealistic in practice. Others have reported very low rates of adherence for online instructional videos (6%) [41] and smart phone applications for bowel preparation (10%) [48], again raising questions about external validity of the study by Prakash, et al. (NB: Reference 48 did not meet inclusion criteria for this review.) More research is needed to examine whether the results of the interventions reviewed here can be replicated in other diverse settings before they can be broadly recommended.

Under the Affordable Care Act, Accountable Care Organizations will face greater pressure to provide cost-effective colorectal cancer screening for their patient populations [49]. This pressure will likely come in the form of increasingly prevalent bundled payments for screening [50] and colonoscopy performance measures [51]. Rates of adequate bowel preparation, adenoma detection and cecal intubation will likely benefit from improved bowel preparation education [3]. In this context, practices considering educational interventions to improve bowel preparation should consider several factors. First, the cost-effectiveness of physician-staffed interventions is likely to be lower than medical assistant-staffed interventions, videos, or brochures. Second, the medium (e.g., online versus printed materials) should ensure accessibility across a full range of age and socioeconomic status. Third, interventions that can be delivered while a patient is present in clinic may be more reliable than interventions that depend on the patient to take additional steps afterward.

The preparation for colonoscopy is more complex, and more important than the preparation for almost any other diagnostic test in medicine. It is therefore incumbent upon gastroenterologists to ensure that all patients undergoing colonoscopy, whether self-referred or open access, receive effective teaching beforehand. While the studies reviewed do not provide definitive guidance on how best to educate patients for colonoscopy, in the opinion of the authors, the first step should be clear and concise written instructions, since every patient likely receives some form of written instructions. Indeed, in six of the seven studies reviewed, the treatment group received standard written instructions along with the patient education intervention.

Healthcare practices may consider adoption of new instructions or review and revision of existing instructions using accepted evaluation tools such as those produced by the Agency for Healthcare Research and Quality (AHRQ) [52]. Ideally high quality written instructions would be combined with an opportunity for verbal in-person or telephone education by an ancillary medical provider, at least for patients at increased risk for preparation failure. When it comes to education about bowel preparation, healthcare practices should recall general principles of health education, including the importance of teach-back, encouraging questions, and hands on demonstration, which are all topics appropriate for review with clinic staff. As noted by AHRQ, “Health materials are effective only when used as part of an overall patient education strategy… If the information is critical, make sure you or someone in your office reviews the information with your patient and/or the patient’s caregiver” [52].

Our review highlights several areas for future research. First, few of the studies that met criteria for inclusion in the review comprehensively assessed known risk factors for colonoscopy preparation failure (e.g., comorbidities, medications, socio-economic status, insurance type, literacy). Future studies should systematically assess these risk factors, which could potentially allow for identification of heterogeneity of treatment effects and lead to targeted approaches that match intervention to selected patient characteristics. Second, only two of the studies included in the review used intention-to-treat analysis, limiting the ability to assess adherence to the interventions and to make conclusions about real-world effectiveness. Third, along the same lines, future studies should systematically assess patient adherence to the bowel preparation, in addition to the education intervention, which will provide further support for efficacy. Fourth, while all three of the studies that used split-dose bowel preparation showed significant effects, others have suggested that educational interventions may not be as efficacious with split dosing [53] (NB: This study used the same brochure as Spiegel, et al. and was published only in abstract form, not meeting criteria for inclusion in this review). Split-dose bowel preparation, which has become standard of care, should be used in future studies of educational interventions. Fifth, future studies would benefit from larger sample sizes with adequate power to detect changes in clinically meaningful outcomes, such as adenoma detection and need for repeat colonoscopy, as well as economic outcomes, such as time and cost [34]. Finally, future studies should carefully consider the advantages and disadvantages of the available bowel preparation scales [54]. For evaluation of patient education interventions to improve bowel preparation, the scale used to measure preparation quality should ideally assess preparation quality before lavage by the endoscopist rather than after, so that the effect of the intervention can be evaluated independently. The scale should also have an established cutoff to distinguish between adequate and inadequate preparation (a dichotomous characterization that is endorsed by GI professional societies but is rarely standardized).

Others have published meta-analyses of patient education interventions to improve bowel preparation [55,56]. Rather than focusing on efforts to address this topic quantitatively with meta-analysis (an approach that we believe is inappropriate in the context of substantial clinical heterogeneity, as highlighted above), our review adds to this existing literature by calling attention to gaps in current knowledge through the lens of health education, discussing important issues of study design for future studies, and helping to contextualize how existing studies, with their limitations, might still be used to improve clinical practice. We would refer readers to the meta-analysis by Guo, et al [56], for a listing of several more recent studies published after the conclusion of our search, which all involved smartphone applications or mobile message reminders, which were intentionally excluded from this systematic review, and the findings of which do not substantially alter our conclusions.

### Limitations

The findings of this review should be interpreted with some caveats. First, because of statistical and clinical heterogeneity, meta-analysis could not be performed. Second, the studies included in the review may be subject to publication bias. We attempted to minimize this risk by searching relevant conference proceedings and including unpublished studies. Third, our results are subject to the same threats to validity that exist in the constituent studies.

### Clinical implications and future directions

This review suggests that several different patient education interventions are efficacious in improving the quality of colonoscopy preparation compared to usual care conditions. These include illustrated brochures, videos, education groups, and re-education phone calls. While none of these interventions has sufficient evidence to recommend broadly, healthcare practices should be aware of these education options as they consider strategies for improving bowel preparation quality. This topic is likely to grow in importance as colonoscopy preparation becomes a greater focus of quality measures and payer reimbursement.

## Supporting Information

S1 File Preferred Reporting Items for Systematic Reviews and Meta-Analyses (PRISMA) checklist. (DOC)

S1 Table Search terms. (DOCX)

S2 Table Scores for Modified Downs and Black Scale for full-text studies included in review. (DOCX)

S3 Table Study descriptions for non-full text studies. (DOCX)

S4 Table Assessment of risk of bias in non-full text studies. (DOCX)

S5 Table Summary of primary outcomes for non-full text studies by preparation scale. (DOCX)
