# Supplementary material for: How Efficacious Are Patient Education Interventions to Improve Bowel Preparation for Colonoscopy? A Systematic Review
Source: PLoS One. 2016 Oct 14;11(10):e0164442. doi: 10.1371/journal.pone.0164442 (PMC5065159; doi:10.1371/journal.pone.0164442)
Supplement: S1 Table — (DOCX) [file pone.0164442.s002.docx]

S1 Table. Search Terms

| **Database** | **Search terms** |
| --- | --- |
| CINAHL | ((MH colonoscopy OR colonoscopy) OR (MH endoscopy OR endoscopy)) AND (MH “patient education” OR “patient education” OR (counsel* OR instruct* OR educat* OR teach*)) AND (MH “bowel preparation” OR MH “cathartics” OR (prep* OR clean* OR cathartic$ OR purgativ*) |
| EMBASE | (‘patient education’/exp OR ‘patient education’ OR counsel* OR instruct* OR teach* OR educat* OR ‘patient education’:ab,ti) AND (‘endoscopy’/de OR ‘intestinal endoscopy’ OR colonoscopy:ab,ti) AND (‘laxative’/de OR ‘x prep’/exp OR cathartic$ OR clean* OR purgativ* OR prep*:ab,ti) |
| Ovid | (purgative$.mp. OR clean$.mp. OR cathartic$.mp. OR prep$.mp. OR exp Cathartics/) AND (educat$.mp. OR instruct$.mp. OR teach$.mp. OR counsel$.mp. OR exp Patient Education as Topic/ OR exp Education/) AND (endoscopy.mp. OR exp Endosscopy/ OR exp Endoscopy, Gastrointestinal/ OR exp Endoscopy, Digestive system/ OR colonoscopy.mp. OR exp Colonoscopy/) |
| Web of Science | ((clean* OR prep * OR cathar* OR purgative*) AND (educat* OR instruct* OR counsel* OR “patient education”) AND (colonoscopy OR endoscopy) |
| Cochrane Database of Systematic Reviews | (endoscopy OR colonoscopy) AND ((patient education) OR counsel* OR instruct* OR educat* OR teach*) AND (bowel preparation OR cathartics OR prep* OR clean* OR cathartic* OR purgative*) |
