# Supplementary material for: How Efficacious Are Patient Education Interventions to Improve Bowel Preparation for Colonoscopy? A Systematic Review
Source: PLoS One. 2016 Oct 14;11(10):e0164442. doi: 10.1371/journal.pone.0164442 (PMC5065159; doi:10.1371/journal.pone.0164442)
Supplement: S2 Table — (DOCX) [file pone.0164442.s003.docx]

S2 Table. Scores for Modified Downs and Black Scale for full-text studies included in review

|  | **Question** | **Calderwood, et al, 2011** | **Hsueh, et al, 2014** | **Liu, et al, 2013** | **Prakash, et al, 2013** | **Shieh, et al, 2013** | **Spiegel, et al, 2011** | **Tae, et al, 2012** |
| --- | --- | --- | --- | --- | --- | --- | --- | --- |
| 1 | Is the hypothesis/aim/objective of the study clearly described? | 1 | 1 | 1 | 1 | 1 | 1 | 1 |
| 2 | Are the main outcomes to be measured clearly described in the intro or methods section? | 1 | 1 | 1 | 1 | 1 | 1 | 1 |
| 3 | Are the characteristics of the patients included in the study clearly described? | 1 | 1 | 1 | 1 | 0 | 1 | 1 |
| 4 | Are the interventions of interest clearly described? | 1 | 0 | 1 | 0 | 1 | 1 | 0 |
| 5 | Are the distributions of principal confounders in each group of subjects to be compared clearly described? (0 if none, 1 if age/gender only, 2 if more) * | 2 | 2 | 2 | 2 | 1 | 2 | 2 |
| 6 | Are the main findings of the study clearly described? | 1 | 1 | 1 | 1 | 1 | 1 | 1 |
| 7 | Does the study provide estimates of the random variability in the data for the main outcomes? | 1 | 1 | 1 | 1 | 1 | 1 | 0 |
| 8 | Have all important adverse events that may be a consequence of the intervention been reported? (1 if cancellations and non-attendance reported, otherwise 0) * | 1 | 1 | 1 | 0 | 0 | 1 | 0 |
| 9 | Have the characteristics of patients lost to follow-up been described? | 0 | 0 | 0 | 0 | 0 | 0 | 0 |
| 10 | Have actual probability values been reported for the main outcomes except where the probability values are less than 0.001? | 1 | 1 | 1 | 1 | 1 | 1 | 1 |
| 11 | Were the subjects asked to participate in the study representative of the entire population from which they were recruited? | 1 | 0 | 1 | 0 | 0 | 1 | 0 |
| 12 | Were those subjects who were prepared to participate representative of the entire population from which they were recruited? | 1 | 0 | 0 | 0 | 0 | 1 | 0 |
| 13 | Were the staff, places, and facilities where the patients were treated, representative of the treatment the majority of patients receive? | 1 | 1 | 1 | 1 | 0 | 1 | 1 |
| 14 | Was an attempt made to blind study subjects to the intervention they have received? | 0 | 0 | 0 | 0 | 0 | 0 | 0 |
| 15 | Was an attempt made to blind those measuring the main outcomes of the intervention? | 1 | 1 | 1 | 1 | 1 | 1 | 1 |
| 16 | If any of the results of the study were based on "data dredging," was this made clear? | 1 | 1 | 1 | 1 | 1 | 1 | 1 |
| 17 | Question excluded from original instrument * | - | - | - | - | - | - | - |
| 18 | Were the statistical tests used to assess the main outcomes appropriate? | 1 | 1 | 1 | 1 | 1 | 1 | 1 |
| 19 | Was compliance with the intervention/s reliable? | 1 | 1 | 1 | 1 | 0 | 1 | 1 |
| 20 | Were the main outcome measures used accurate (valid and reliable)? | 1 | 1 | 1 | 1 | 1 | 1 | 1 |
| 21 | Were the patients in different intervention groups (trials and cohort studies) or were the cases and controls (case-control studies) recruited from the same population? | 1 | 1 | 1 | 1 | 1 | 1 | 1 |
| 22 | Were study subjects in different intervention groups (trials and cohort studies) or were the cases and controls (case-control studies) recruited over the same period of time? | 1 | 1 | 1 | 1 | 1 | 1 | 1 |
| 23 | Were study subjects randomized to intervention groups? | 1 | 0 | 1 | 1 | 0 | 1 | 1 |
| 24 | Was the randomized intervention assignment concealed from both patients and health care staff until recruitment was complete and irrevocable? | 0 | 0 | 1 | 1 | 0 | 0 | 0 |
| 25 | Was there adequate adjustment for confounding in the analyses from which the main findings were drawn? | 0 | 0 | 1 | 0 | 0 | 1 | 0 |
| 26 | Were losses of patients to follow-up taken into account? | 0 | 0 | 1 | 0 | 0 | 1 | 0 |
| 27 | Did the study have sufficient power to detect a  clinically important effect where the probability  value for a difference being due to chance is  less than 5%?  (1 if yes, 0 if no) | 1 | 0 | 1 | 0 | 0 | 1 | 1 |

*Question was modified from original instrument
