# Supplementary material for: How Efficacious Are Patient Education Interventions to Improve Bowel Preparation for Colonoscopy? A Systematic Review
Source: PLoS One. 2016 Oct 14;11(10):e0164442. doi: 10.1371/journal.pone.0164442 (PMC5065159; doi:10.1371/journal.pone.0164442)
Supplement: S3 Table — (DOCX) [file pone.0164442.s004.docx]

S3 Table. Study descriptions for non-full text studies

| **Study, year** | **Date and location** | **Treatment intervention** | **Control intervention** | **Bowel preparation used*** | **Staffing** | **Colonoscopy indication** | **Patients analyzed (n intervention / n control)** |
| --- | --- | --- | --- | --- | --- | --- | --- |
| Bowman, et al, 2014 [40]] | Date not stated, US | Educational video (not stated if delivered online or in-person), designed with patient education specialists | Standard instructions | PEG, majority split dose | N/A | Screening or surveillance | 179/171 |
| Ergen, et al, 2014 [44] | US, 2013 | Educational brochure used by Spiegel et al [34], adapted to inpatient setting | Standard inpatient bowel preparation | 4L PEG, split dose | N/A | Diagnostic | 43/40 |
| Kakkar, et al, 2013 [41] | US, 2010-2011 | On-line five-minute educational video covering screening rationale, bowel preparation, and diet, with pictures of prepared and unprepared colons. Mailed postcard and phone reminder directed patients to website | Standard written instructions | Bisacodyl + 4L PEG, split dose for afternoon procedures | N/A | Not limited | 387/350 |
| Pillai, et al, 2013 [42] | Date not stated, US | 6-minute instructional video on colonoscopy | Instructional video on gastroesophageal reflux | Not stated | N/A | Screening | 56/48 |
| Yun, et al, 2014 [43] | Not stated | “Photo instructions” | Written instructions | Not stated | Not stated | Not stated | 79/93 |

*If not stated in table, the study did not specify whether split dose was used
