# Supplementary material for: How Efficacious Are Patient Education Interventions to Improve Bowel Preparation for Colonoscopy? A Systematic Review
Source: PLoS One. 2016 Oct 14;11(10):e0164442. doi: 10.1371/journal.pone.0164442 (PMC5065159; doi:10.1371/journal.pone.0164442)
Supplement: S4 Table — (DOCX) [file pone.0164442.s005.docx]

S4 Table. Assessment of risk of bias in non-full text studies*

| **Study, year** | **Allocation method** | **Endoscopist blinding** | **Intention-to-treat analysis** |
| --- | --- | --- | --- |
| Bowman, et al, 2014 [40] | Randomization | Yes | No |
| Ergen, et al, 2014 [44] | Randomization | Yes | No |
| Kakkar, et al, 2013 [41] | Randomization | Yes | No |
| Pillai, et al, 2013 [42] | Randomization | Yes | Not stated |
| Yun, et al, 2014 [43] | Randomization | Yes | Not stated |

* Study validity score could be not be calculated because of limited available information on study characteristics
