# Supplementary material for: How Efficacious Are Patient Education Interventions to Improve Bowel Preparation for Colonoscopy? A Systematic Review
Source: PLoS One. 2016 Oct 14;11(10):e0164442. doi: 10.1371/journal.pone.0164442 (PMC5065159; doi:10.1371/journal.pone.0164442)
Supplement: S5 Table — (DOCX) [file pone.0164442.s006.docx]

S5 Table. Summary of primary outcomes for non-full text studies by preparation scale

| **Study, year** | **Ottawa Bowel Preparation Scale ^*^** | | **Boston Bowel Preparation Scale ^%^** | | **Aronchick Bowel Preparation Scale ^$^** | |
| --- | --- | --- | --- | --- | --- | --- |
|  | **Outcome** | **p-value** | **Outcome** | **p-value** | **Outcome** | **p value** |
| Bowman, et al, 2014 [40] |  |  | Score >6:  Intervention: 74.9%  Control: 64.9% | 0.042 |  |  |
| Ergen, et al, 2014 [44] |  |  | Total score >6 and all bowel segments >2:  Intervention: 60%  Control: 35% | 0.02 |  |  |
| Kakkar, et al, 2013 [41] |  |  | All segments > 2:  Intervention: 88%  Control: 89%^#^ | 0.85 |  |  |
| Pillai, et al, 2013 [42] | Excellent bowel prep (not further defined):  Intervention: 53%  Control: 31% | <0.01 |  |  |  |  |
| Yun, et al, 2014 [43] | Mean (sd):  Intervention: 6.1 (2.9)  Control: 5.0 (3.1) | 0.013 |  |  |  |  |

*Scale ranges from 14 (very poor) to 0 (excellent)

^%^Scale ranges from 0 (very poor) to 9 (excellent)

^$^Likert scale ranges from “excellent” to “inadequate”

^#^Authors also summarized outcomes with group medians and/or means
